# Three-dimensional morphological analysis of 67 primary mandibular first molars based on CBCT reconstructions

**DOI:** 10.1186/s12903-026-09384-2

**Published:** 2026-07-23

**Authors:** Kefan Li, Jie Bai, Aonan Wen, Yijiao Zhao, Runkai Wang, Tianyu Ma, Bin Xia

**Affiliations:** 1 Department of Pediatric Dentistry, Digital Medical Devices & Research Center of Engineering and Technology for Digital Dentistry of Ministry of Health & Beijing Key Laboratory of Digital Stomatology & National Clinical Research Center for Oral Diseases, Peking University School and Hospital of Stomatology & National Engineering Research Center of Oral Biomaterials, 22 South Avenue Zhongguancun, Haidian District, Beijing, 100081 China; 2 Department of Oral Emergency, Digital Medical Devices & Research Center of Engineering and Technology for Digital Dentistry of Ministry of Health & Beijing Key Laboratory of Digital Stomatology & National Clinical Research Center for Oral Diseases, Peking University School and Hospital of Stomatology & National Engineering Research Center of Oral Biomaterials, Beijing, 100081 China; 3 Centre of Digital Dentistry, National Center for Stomatology & National Clinical Research Center for Oral Diseases & National Engineering Research Center of Oral Biomaterials and Digital Medical Devices & Beijing Key Laboratory of Digital Stomatology & NHC Research Center of Engineering and Technology for Computerized Dentistry, Peking University School and Hospital of Stomatology, Beijing, 100081 China

**Keywords:** PMFMs, Cone-beam computed tomography, Three-dimensional reconstruction, Anatomical variation, Morphometry, Root canal treatment

## Abstract

**Background:**

Despite previous anatomical studies on PMFMs, detailed classification and comprehensive measurements remain limited, and the association between crown and root morphology has not been systematically investigated. This study aims to reconstruct three-dimensional anatomical models of PMFMs from intact CBCT images obtained from children in Beijing, China, to characterize variations in crown and root morphology and their relationships, and to conduct detailed quantitative and statistical analyses based on refined classifications.

**Methods:**

Three-dimensional (3D) anatomical models of 67 intact PMFMs were reconstructed from CBCT scans using a semi-automatic threshold-based region segmentation method. Key morphological parameters of the crown and root were measured. Samples were primarily classified based on the ratio of mesiodistal to buccolingual crown diameter (R) and the number of roots (N), respectively, and their association was explored. A secondary classification was conducted by incorporating the number of root canals (n). For subgroups with sufficient sample sizes, detailed morphological parameters were measured and compared among types.

**Results:**

Two crown variants were identified. *R* > 1 was observed in 59 teeth (88.1%), with a mean value of 1.11 ± 0.06, while *R* < 1 was observed in 8 teeth (11.9%), with a mean of 0.94 ± 0.03. A significant difference was found between the R values (*p* < 0.05). Two root number variants were identified. Fifty-eight teeth (86.6%) had two mesiodistally aligned roots, all with *R* > 1. Nine teeth (13.4%) had three roots, in which 8 (11.9%) had *R* < 1; and 1 (1.5%) had *R* > 1. A significant negative correlation was found between R and N (*r* = -0.61, *p* < 0.05), and ROC curve analysis confirmed that *R* = 1.00 was the optimal cutoff for distinguishing two-rooted from three-rooted PMFMs (AUC = 1.000). Besides, a secondary classification identified seven types, among which the mesiobuccal canal was consistently the longest (*p* < 0.05).

**Conclusion:**

PMFMs exhibit considerable morphological variations. The ratio of mesiodistal to buccolingual crown diameter (R) is a useful predictor of root number, and the mesiobuccal canal is significantly longer than the other canals. These features may aid clinicians in preoperative planning.

## Introduction

Primary molars have a crucial role in pediatric dentition, contributing to masticatory function, promoting jaw development, and maintaining space for the eruption of permanent teeth. Epidemiological surveys indicate that PMFMs have the highest caries incidence among 5-year-old children [1]. Dental caries can compromise crown morphology and, if untreated, may progress to pulpitis and periapical periodontitis [2–4]. Successful crown restoration and endodontic treatment rely on a thorough understanding of tooth anatomy. Previous studies have established a fundamental anatomical framework for PMFMs; however, due to physiological root resorption and a high caries rate of PMFMs, obtaining intact extracted PMFMs samples with complete crowns and roots is extremely challenging, which has historically limited comprehensive morphological studies [5–10]. 

Previous studies have described the unique anatomical morphology of PMFM crowns. Based on 3D intraoral scans, Chao et al. [10] identified multiple variations in PMFM crown morphology, including rhomboid, triangular, square, or rectangular. Although several studies have reported crown measurements, the results vary considerably, possibly due to ethnic differences or inconsistent measurement landmarks [11–13]. This variability underscores the need for updated PMFMs crown data using standardized and reproducible measuring methods. Regarding root and canal morphology, PMFMs are generally described as two-rooted, with three-rooted cases reported less frequently and with variable incidence [6, 16]. The number of canals has been reported to range from 3 to 4. Several reports have provided detailed measurements of root or canal lengths; however, to our knowledge, no study has systematically measured the full working length of PMFMs [5–9, 14–16].

Since intact extracted PMFMs are difficult to obtain, previous anatomical studies have largely focused on either crown or root morphology separately, and the potential association between these two aspects has received limited attention. To date, only Moyaho-Bernal et al. [16] have suggested a possible link, reporting that three-rooted PMFMs had crowns characterized by a distolingual lobe and a cervical prominence, with the additional root located lingually. However, no quantitative anatomical evidence has been provided to support this observation.

In recent years, the widespread use of CBCT in pediatric dentistry has enabled the acquisition of 3D data on healthy, intact PMFMs in young children, facilitating more comprehensive anatomical studies [6, 7, 11, 17–19]. CBCT scans acquired for diagnostic purposes in cases such as dental trauma or supernumerary teeth provide access to sufficient data for analysis [18, 20]. Digital techniques enable the reconstruction of 3D models from CBCT images, facilitating the identification of anatomical details that are difficult to appreciate in cross-sectional views and allowing multi-dimensional measurements [20, 21]. Furthermore, Li et al. demonstrated, using micro-CT as the gold standard, the accuracy of CBCT-derived 3D reconstruction models for anatomical studies [22]. Therefore, the use of 3D reconstruction models derived from clinical CBCT databases offers a promising approach for advancing anatomical studies of PMFMs.

This study aimed to reconstruct 3D anatomical models of PMFMs from a relatively large sample of intact CBCT images obtained from children in Beijing, China, to characterize crown and root morphological variations and their associations, and to perform quantitative and statistical analyses under detailed classification.

## Materials and methods

### Data collection

CBCT data were collected from children aged 3–5 years who attended Peking University Hospital of Stomatology between January 2019 and April 2022. All CBCT scans were acquired for clinical diagnosis and treatment purposes unrelated to the present study. Eligible CBCT datasets were stored in Digital Imaging and Communications in Medicine (DICOM) format. All procedures complied with the relevant laws and institutional guidelines [23]. The study was approved by the Ethics Committee (Ethics Approval Number: PKUSSIRB-202281147). The requirement for informed consent was waived due to the retrospective nature of the study and the use of anonymized data, which posed no additional risk to participants.

### Inclusion/exclusion criteria

Inclusion criteria: CBCT scans with clear, intact images of PMFMs; absence of morphological anomalies; no hard tissue defects or caries extending into the dentine layer; complete apical development without significant internal or external resorption; and no evidence of previous treatment.

Exclusion criteria: CBCT scans with substantial motion artifacts.

### Sample size calculation

A pilot study was conducted, and sample size estimation was performed using PASS 2021 (NCSS LLC., Kaysville, UT., USA) based on the maximum observed standard deviation of mesiodistal and buccolingual crown diameters. Assuming a significance level of α = 0.05 and a margin of error of ± 0.5 mm, the required sample size was estimated to be five specimens per variant.

### Radiographic techniques

Images were acquired using a 3D Accuitomo CBCT unit (J. Morita Manufacturing Corp., Kyoto, Japan) with a voxel size of 0.125 mm. All scans were performed by licensed radiologists at 90 kV and 5.0 mA, with an exposure time of 17.5 s.

### Sample model reconstruction and analysis

#### 3D modelling

**Fig. 1:**
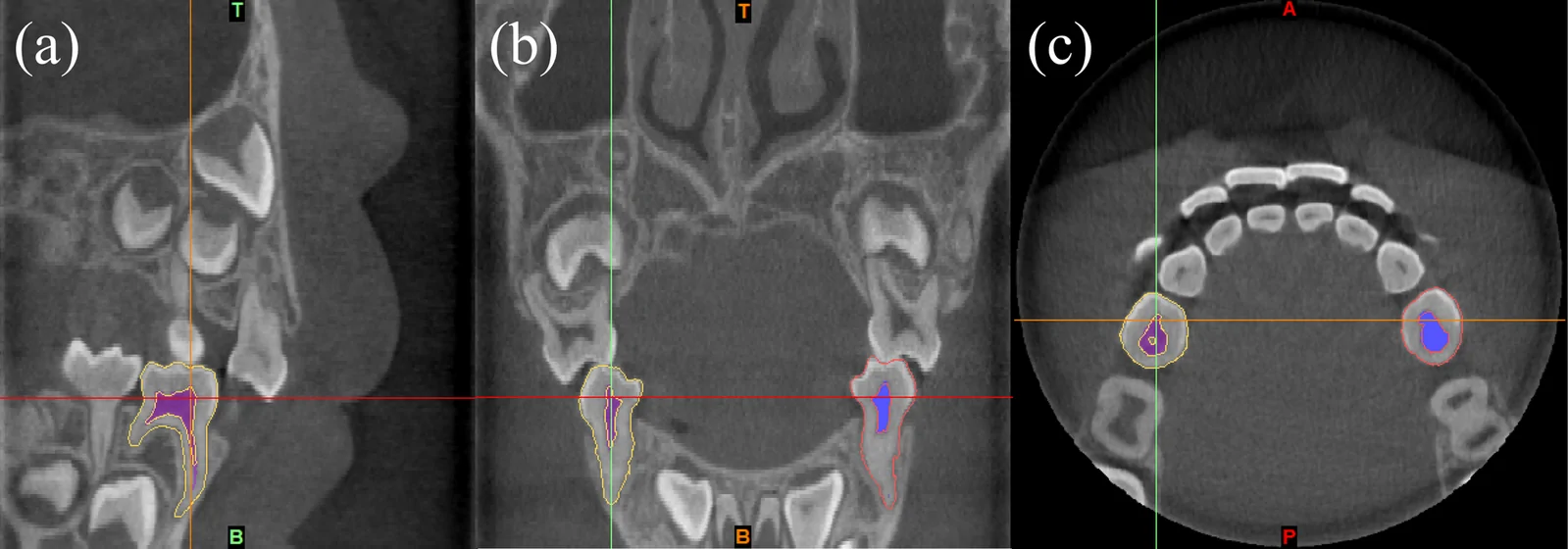
Segmentation of PMFMs. **a** Buccolingual cross-section of a right PMFM; **b** Mesiodistal cross-sections of bilateral PMFMs; **c** Cross-sectional slices of bilateral PMFMs. Yellow or red outlines were segmentation results based on hard tissue threshold; purple or blue areas were pulp regions corrected by the clinician

Three-dimensional (3D) models of the hard tissues and pulp cavity of PMFMs were generated using a threshold-based semi-automatic segmentation method in Mimics 24.0 (Materialise NV, Leuven, Belgium). The PMFM segments were reviewed by two paediatric dentists (J.B. and B.X.) with over 20–30 years of clinical experience, and manually refined when the threshold-based segmentation was suboptimal, to ensure that the resulting models were consistent with the original CBCT. The refined models were then exported in ‘.stl’ format. In the case of differing opinions between the two experts, consensus was reached after discussion (Fig. 1). To evaluate the reproducibility of the segmentation protocol, one operator repeated the entire segmentation process on 20 randomly selected teeth after a one-month interval. The intraclass correlation coefficient (ICC) for the repeated volume measurements was 0.95, indicating excellent reproducibility.

#### Coordinate adjustment

**Fig. 2:**
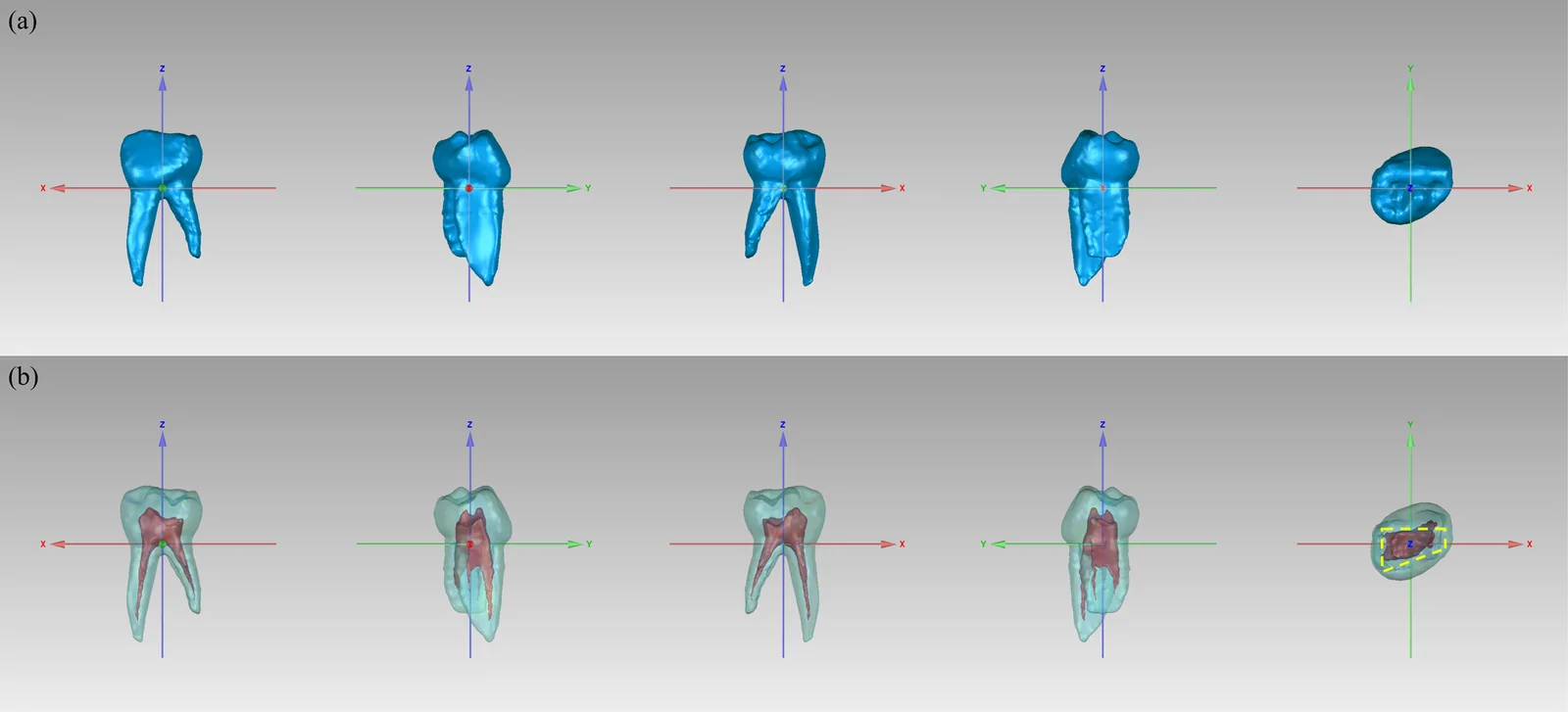
Type A two-rooted PMFM, with mesial and distal roots, each containing two canals distributed buccolingually. From left to right: Buccal view, mesial view, lingual view, distal view, occlusal view. **a** Hard tissue external surface; **b** corresponding transparent view showing the position of pulp. In the occlusal view of the transparent model (**b**), the dashed lines indicate the trapezoidal crown contour, which can be compared with the solid occlusal view in (**a**), and allows direct comparison between the crown margin and the underlying pulp chamber and canal configuration

**Fig. 3:**
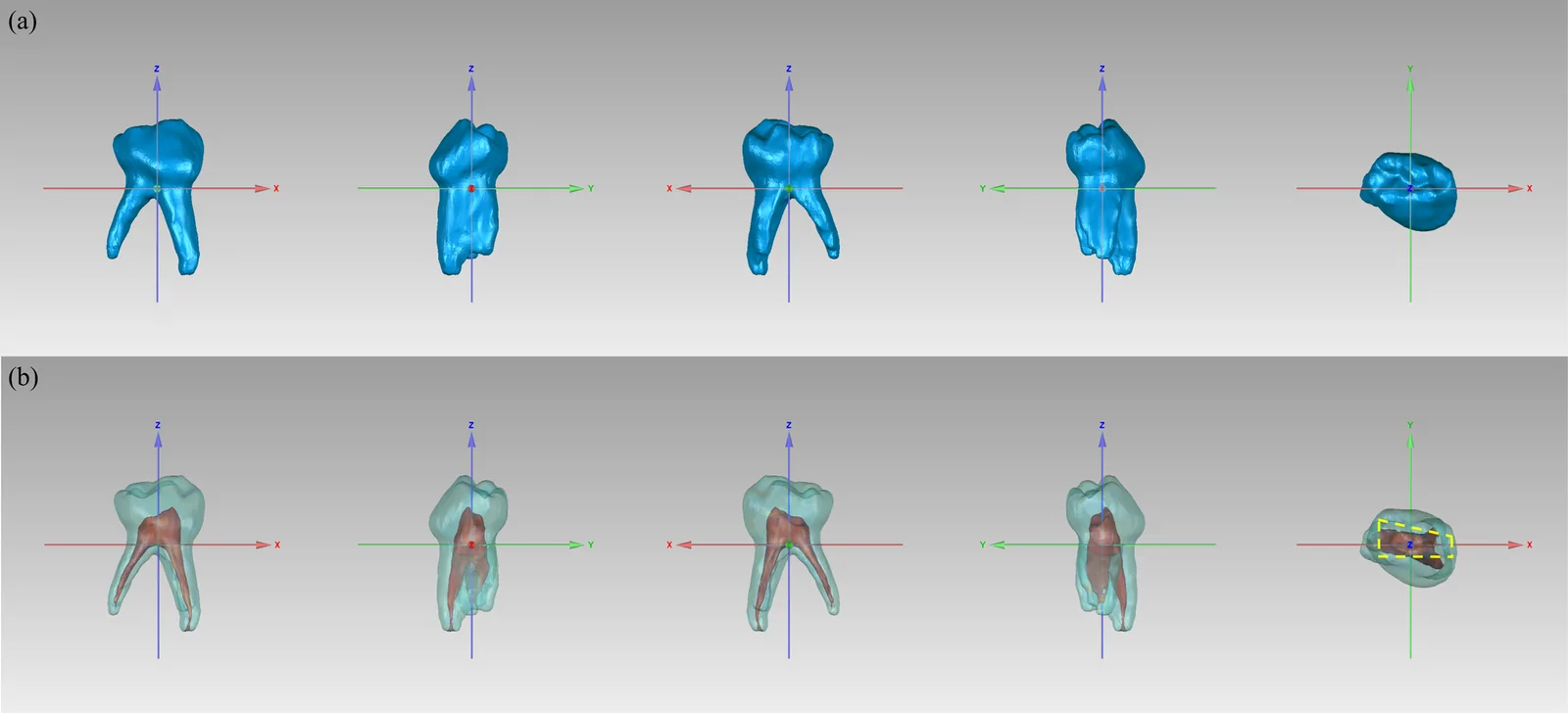
Type B two-rooted PMFM, with the mesial root containing two buccolingually arranged canals, and the distal root containing one canal. Views and layout are the same as in Fig. 2. **a** Hard tissue external surface; **b** corresponding transparent view showing the position of pulp. The dashed lines indicate the trapezoidal crown contour

**Fig. 4:**
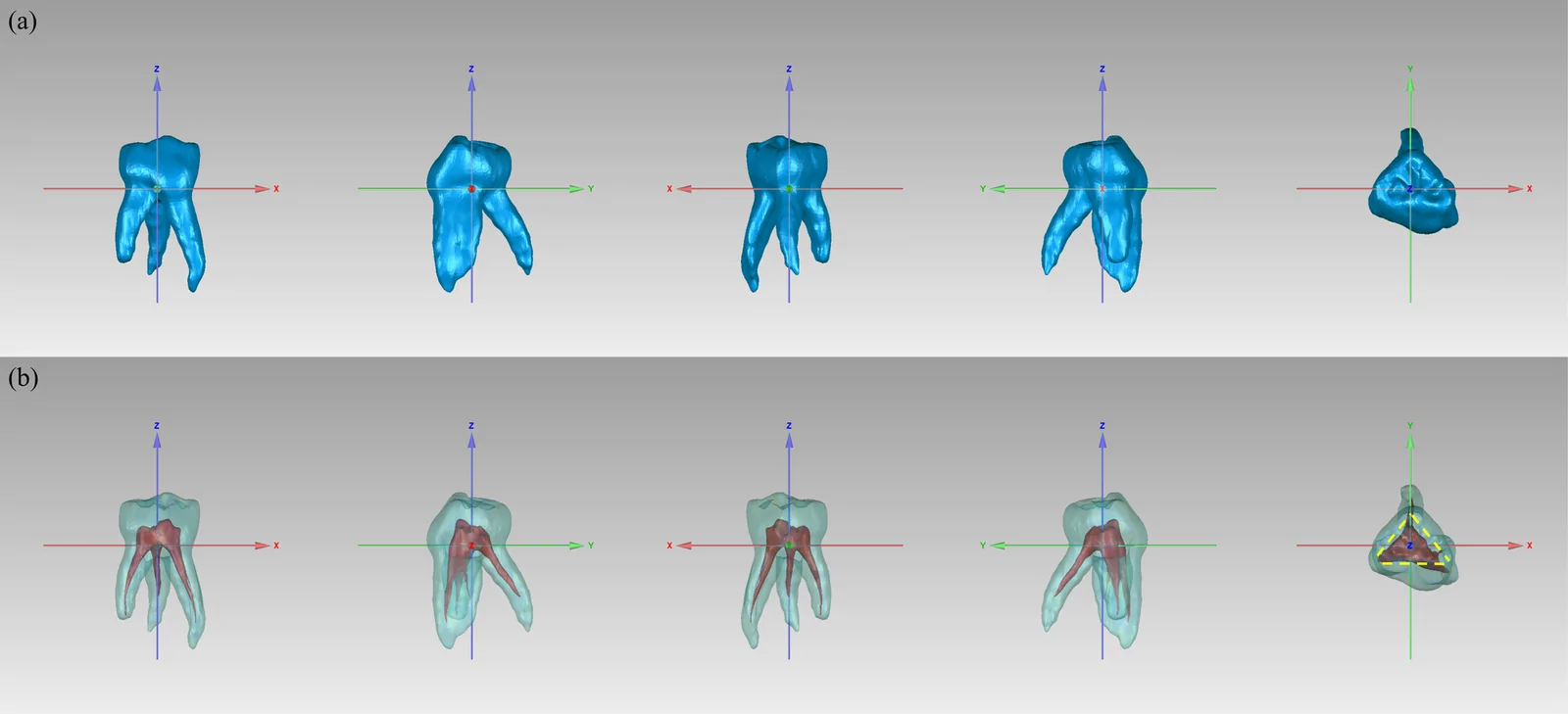
Type D three-rooted PMFMs, with mesial, distal, and lingual roots, the mesial root containing two buccolingually arranged canals, and the distal and lingual roots each containing a single canal. Views and layout are the same as in Fig. 2. **a** Hard tissue external surface; **b** corresponding transparent view showing the position of pulp. The dashed lines indicate the triangular crown contour

The coordinate axes of the models were adjusted using Geomagic Control X (3D Systems Corp., Rock Hill, SC, USA). For all PMFMs, the midpoint of the pulp chamber floor was set as the coordinate origin. The models were then oriented such that the cervical line was parallel to the y-axis in the distal view and the x-axis in the lingual view. In the occlusal view, the line connecting the mesiobuccal and distobuccal cusp tips was aligned parallel to the x-axis. Consequently, the z-axis corresponded to the long axis of the tooth (Figs. 2, 3 and 4). A preliminary experiment showed that inter-observer agreement based on measurements performed according to this standard was excellent, with an intraclass correlation coefficient (ICC) of 0.96.

#### Variant type classification

The models were evaluated in the coronal, sagittal, and axial views by a licensed pediatric dentist. Crown variation was primarily characterized by the ratio of mesiodistal to buccolingual diameter (R; see "Measurement of morphological parameters" section). The primary classification was performed independently based on the crown ratio (R) and root number (N), respectively, and the association between R and N was explored. Subsequently, a refined secondary classification was conducted by further integrating the characteristics of R, N, as well as the number of root canals per root (n). The classification results were independently reviewed by a pediatric specialist with over 30 years of clinical experience (B.X.) and four additional licensed pediatric dentists (K.L., J.B., R.W., T.M.). All observers were trained in morphological classification and passed the relevant examinations. Subsequently, a pilot study was conducted to perform a standard consistency test on results of tooth anatomy classifications by the observers. The Fleiss Kappa coefficient was 0.924 for inter-observer agreement, and the mean Cohen’s Kappa was 0.942-1 for intra-observer reliability when reassessed after 1 month.

#### Anatomical detail observation of the roots

Root morphology was assessed for the presence, direction, and location of curvature. Canal morphology was evaluated for the presence of lateral or accessory canals, isthmuses, and apical deltas.

#### Measurement of morphological parameters

**Fig. 5:**
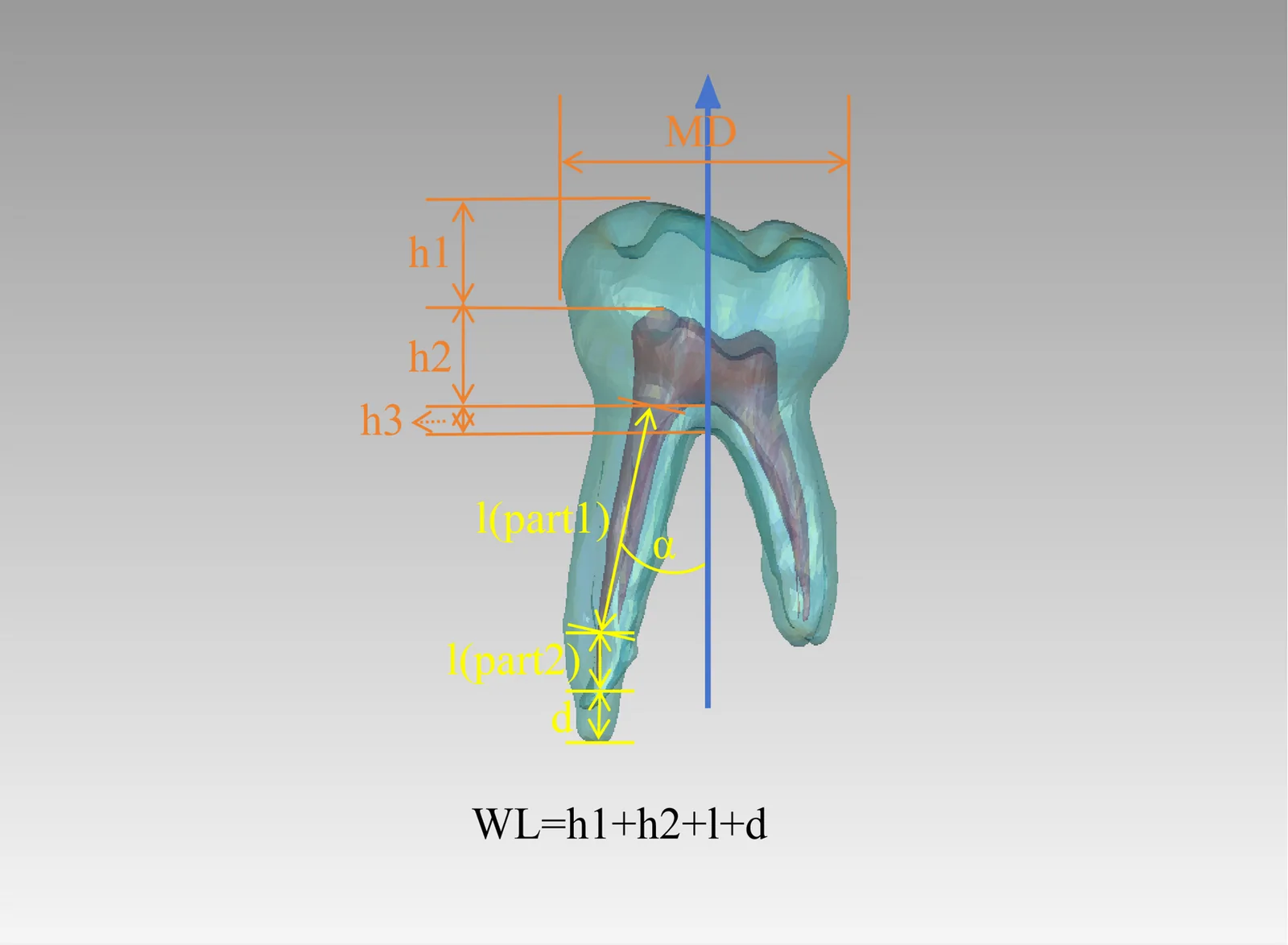
Illustration of length and angle measurements for a PMFM. Crown measurements are indicated in brown, and root measurements in yellow. In this specimen, the root canal is curved; therefore, the observable canal length (l) was measured in two segments: l(part1) and l(part2)

A standardized and reproducible measurement protocol was established for all PMFMs (Fig. 5).

Along the x, y, and z axes, the following crown parameters were measured: mesiodistal diameter (MD), buccolingual diameter (BL), and crown height (H). The ratio R (MD/BL) was subsequently calculated.

Along the z-axis, the following vertical distances were measured: from the highest point of the pulp chamber roof to the occlusal surface (h1), from the highest point of the pulp chamber roof to the midpoint of the pulp chamber floor (h2), and from the midpoint of the pulp chamber floor to the midpoint of the furcation (h3).

The angle (α) between each root and the z-axis was measured.

The distance from each canal orifice to the canal terminus was measured and defined as the observable canal length (l). In canals with pronounced curvature, the lengths of individual segments were measured separately and summed. The distance (d) from the canal terminus to the anatomical root apex was also recorded. The predicted canal length (L) was calculated as L = l + d, and the total working length (WL) was calculated as WL = h1 + h2 + l + d.

#### Statistical analysis

Statistical analyses were performed using SPSS Statistics 26.0 (IBM Corp., Armonk, NY, USA).

In the primary classification, normality of R was assessed using the Kolmogorov-Smirnov (K-S) test, and homogeneity of variance was evaluated. If the data were normally distributed with homogeneous variance, comparisons were performed using the independent samples t-test; otherwise, the Mann-Whitney U test was used. Spearman’s correlation analysis was used to assess the relationship between R and N. In addition, receiver operating characteristic (ROC) curve analysis was performed to evaluate the discriminative ability of the R value for identifying N. The area under the curve (AUC), sensitivity, and specificity were calculated, and the optimal cutoff value was determined using the Youden index.

To account for the potential non-independence of observations due to bilateral teeth from the same patient, a generalized linear mixed model (GLMM) with anonymous patient code as a random effect using a binary logistic structure was fitted with three-root presence as the dependent variable and R value as the fixed effect, to adjust for patient-level clustering, and estimate the random effect variance to assess the magnitude of clustering.

Within each secondary classification, comparisons were conducted based on side (left vs. right), sex, and variant type. The normality of each parameter was assessed using the K-S test, and homogeneity of variance was evaluated. If the data were normally distributed with homogeneous variance, comparisons were performed using the independent samples t-test; otherwise, the Mann-Whitney U test was applied.

All tests were two-tailed, and the significance level was α = 0.05. Bonferroni correction was applied for all multiple comparisons.

Effect sizes for group comparisons were evaluated using Cohen’s d, with values of 0.20, 0.50, and 0.80 interpreted as small, medium, and large effects, respectively.

## Results

A total of 41 CBCT datasets (25 males, 16 females; mean age 3.86 years) were included. Sixty-seven PMFMs were analyzed, including 35 left and 32 right teeth.

### Classification and statistical analysis of primary variation types

Two main crown morphological variants were identified in PMFMs. The first variant (*R* > 1) exhibited a trapezoidal outline in the occlusal view, formed by the mesial and distal marginal ridges, and the line connecting the buccal and lingual cusps. The lingual surface was straight, and the cervical line was approximately linear. This variant included 59 teeth (88.1%). The second variant (*R* < 1) exhibited a triangular occlusal outline. The lingual surface was convex, extending toward the lingual root, and the cervical margin also showed a triangular configuration. This variant included 8 teeth (11.9%) (Figs. 2, 3 and 4, see "Classification and statistical analysis of secondary variation types" section for details). The mean R value was 1.11 ± 0.06 for the *R* > 1 group, and 0.94 ± 0.03 for the *R* < 1 group, with a significant difference between groups (*p* < 0.05; Cohen’s d = 2.34, indicating a large effect).

Two root number variants were identified. Fifty-eight teeth (86.6%) had two mesiodistally aligned roots, all with *R* > 1. Nine teeth (13.4%) had three roots, in which 8 (11.9%) had mesial, distal, and lingual roots, all with *R* < 1; this variant was bilateral in 3 of 4 patients (75%). One (1.5%) had mesiobuccal, mesiolingual, and distal roots, with *R* > 1.

**Fig. 6:**
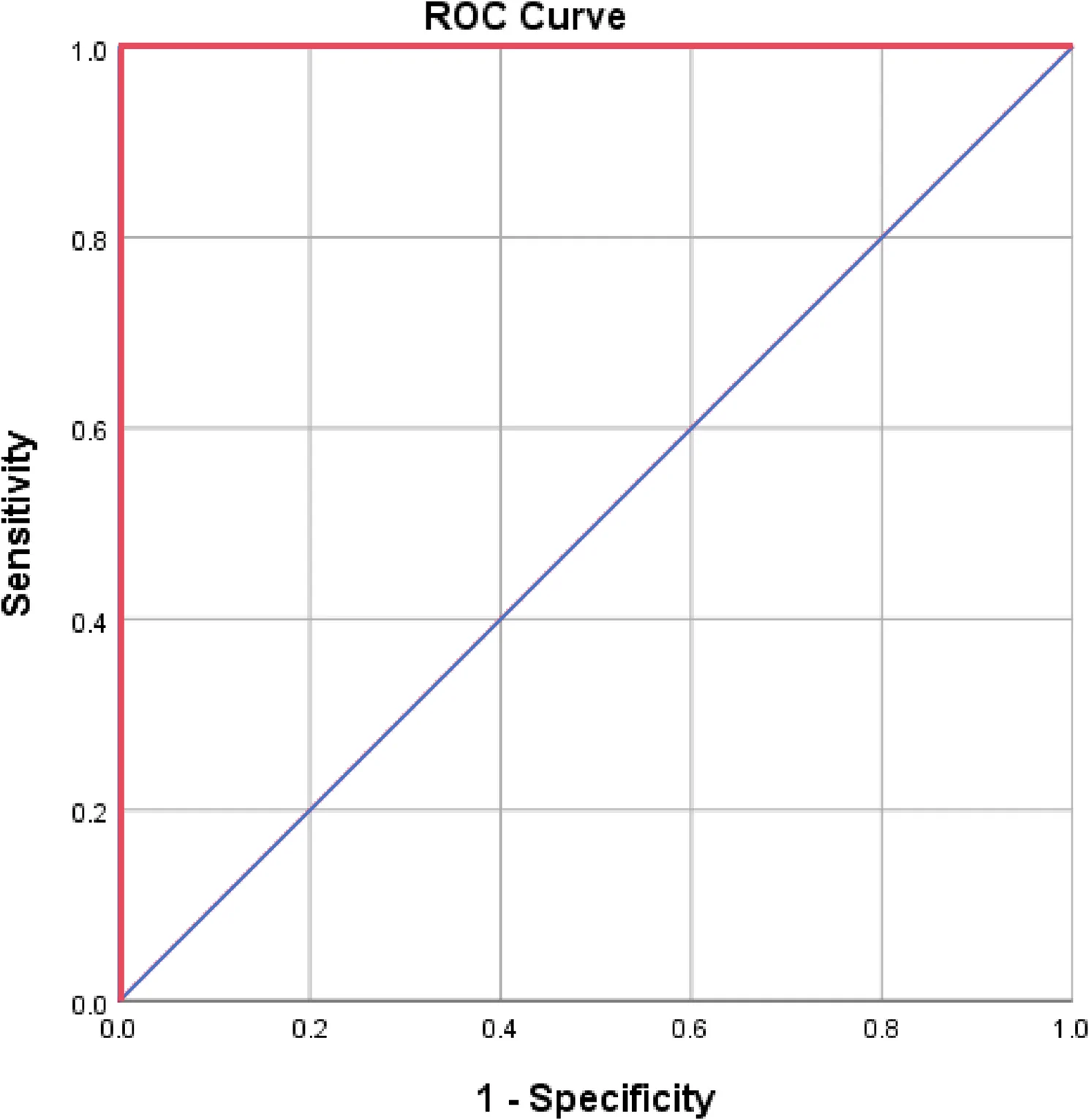
ROC curve confirming *R* = 1.00 as the optimal cutoff for distinguishing two-rooted from three-rooted teeth (AUC = 1.00, *p* < 0.05)

Statistical analysis revealed a significant negative correlation between R and N (*r* = -0.61, *p* < 0.05), indicating that higher R values were associated with fewer roots. In addition, ROC curve analysis confirmed that the R value could perfectly distinguish two-rooted from three-rooted teeth, with an optimal cutoff of *R* = 1.00 (AUC = 1.00, *p* < 0.05) (Fig. 6).

Furthermore, A GLMM with anonymous patient code as a random effect confirmed that the association between R value and root number remained robust after adjusting for patient-level clustering (*p* < 0.05), with negligible clustering variance.

### Classification and statistical analysis of secondary variation types

PMFMs were further classified based on root canal variations, and seven types were identified. Types A-C were associated with *R* > 1, whereas types D-D1 were associated with *R* < 1.

Type A: 42 teeth (62.7%), with mesial and distal roots, each containing two canals distributed buccolingually (Fig. 2).

Types A1 and A2: 4 specimen of this type were observed., similar to Type A, but with an additional accessory canal branching from the upper portion of one canal in either the mesial or distal root, or between two canals within the same root.

Type B: 12 teeth (17.9%), with two roots; the mesial root contained two buccolingually arranged canals, and the distal root contained one canal (Fig. 3).

**Fig. 7:**
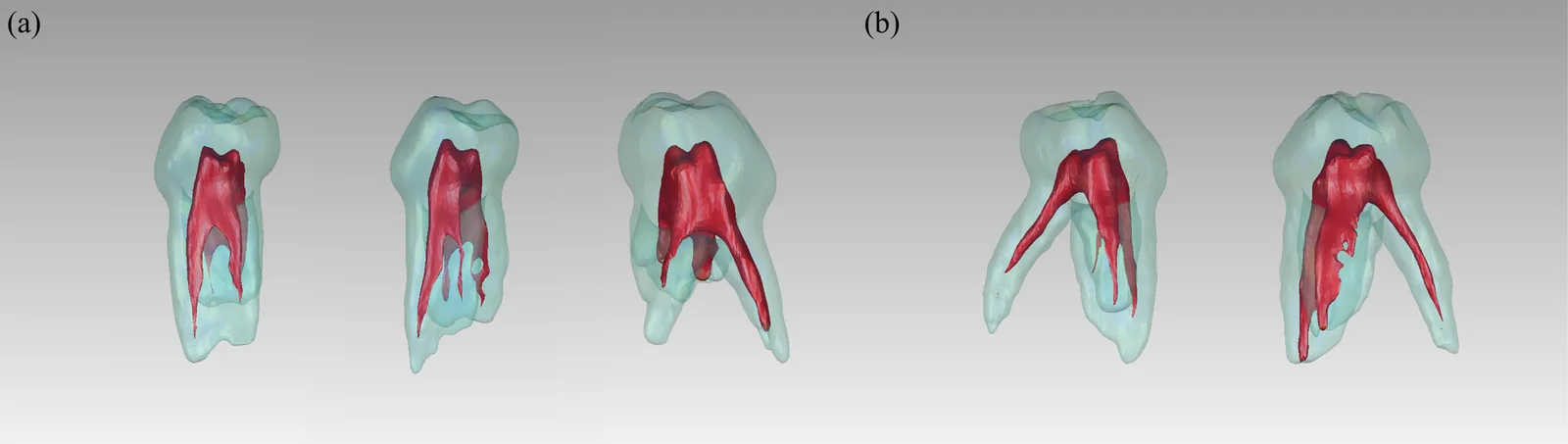
Comparison of PMFMs with those having the same crown variation. **a** Comparison of Type A (left), Type A1 (middle), Type C (right); **b** Comparison of Type D (left) and Type D1 (right)

Type C: 1 specimen of this type was observed., with three roots (mesiobuccal, mesiolingual, and distal). Each mesial root contained one canal, while the distal root contained two canals (Fig. 7).

Although the number of canals varied among types A-C, their canal orifices were consistently located on the mesial and distal aspects of the pulp chamber floor along the buccolingual direction. The lines connecting the orifices within each root were approximately parallel to the y-axis and perpendicular to the line connecting the mesiobuccal and distobuccal cusps in the occlusal view.

Type D: 7 teeth (10.4%), with three roots (mesial, distal, and lingual). The mesial root contained two buccolingually arranged canals, whereas the distal and lingual roots each contained a single canal (Fig. 4).

Type D1: 1 specimen of this type was observed, with similar features to Type D, but with an additional accessory canal on the lingual side of the distal root (Fig. 7).

In types D and D1, canal orifices in the mesial and distal roots were located on the corresponding aspects of the pulp chamber floor along the buccolingual direction. The lines connecting the orifices within these roots were approximately parallel to the y-axis and perpendicular to the line connecting the mesiobuccal and distobuccal cusps in the occlusal view. The orifice of the lingual canal was typically located at the most lingual aspect of the midpoint of the pulp chamber floor.

As Type A PMFMs represented the largest proportion, their measurement data were used as a baseline for comparison with other types.

The mean mesiodistal diameter of Type A PMFMs was 8.61 ± 0.42 mm, and the buccolingual diameter was 7.77 ± 0.31 mm, yielding a mean ratio (R) of 1.11 ± 0.06 (Table 1). No significant differences were observed between Types A and B for these parameters (*p* > 0.05) (Tables 1 and 2). The mesiodistal diameter of Type D did not differ significantly from that of Type A; however, the buccolingual diameter was significantly greater (8.86 ± 0.36 mm) than in Types A (*p* < 0.05,Cohen’s d = 2.53), and the R value was < 1, significantly lower than that of Type A (*p* < 0.05, Cohen’s d = 2.51) (Tables 1 and 3). The mean crown height of Type A PMFMs was 6.64 ± 0.33 mm, with no significant differences compared to Types B and D (*p* > 0.05) (Tables 1, 2 and 3).

**Table 1:** Measurements of Type A PMFMs

Type A	Mean	Standard deviation	Max	Min	Median	95% CI
MD (mm)	8.61	0.42	9.59	7.80	8.66	(7.79, 9.43)
BL (mm)	7.77	0.31	8.55	7.16	7.81	(7.16, 8.38)
R	1.11	0.06	1.27	1.01	1.10	(0.99, 1.23)
H (mm)	6.64	0.33	7.44	6.12	6.65	(5.99, 7.29)
h1 (mm)	2.48	0.27	2.99	1.91	2.53	(1.95, 3.01)
h2 (mm)	3.27	0.48	4.28	2.43	3.15	(2.33, 4.21)
h3 (mm)	1.16	0.23	1.95	0.88	1.11	(0.71, 1.61)
α(M) (°)	15.82	3.49	23.02	9.52	14.89	(8.98, 22.66)
α(D) (°)	23.51	3.21	31.09	15.67	22.66	(17.22, 29.80)
l(mb) (mm)	8.32	1.06	10.12	6.26	8.57	(6.24, 10.40)
d(mb) (mm)	1.45	0.51	2.73	0.00	1.45	(0.45, 2.45)
L(MB) (mm)	9.77	1.12	11.90	7.54	10.04	(7.57, 11.97)
WL(MB) (mm)	15.52	1.05	17.24	13.53	15.79	(13.46, 17.58)
l(ml) (mm)	5.25	1.07	7.47	3.16	5.20	(3.15, 7.35)
d(ml) (mm)	1.81	0.62	2.74	0.33	1.99	(0.59, 3.03)
L(ML) (mm)	7.06	1.12	8.80	4.61	7.26	(4.86, 9.26)
WL(ML) (mm)	12.81	1.06	14.53	10.42	12.74	(10.73, 14.89)
l(db) (mm)	5.80	1.01	7.67	3.61	5.78	(3.82, 7.78)
d(db) (mm)	0.90	0.54	1.87	0.00	0.98	(0, 1.96)
L(DB) (mm)	6.70	0.99	9.12	4.65	6.92	(4.76, 8.64)
WL(DB) (mm)	12.45	1.05	14.96	10.21	12.66	(10.39, 14.51)
l(dl) (mm)	5.63	1.13	7.67	3.04	5.87	(3.42, 7.84)
d(dl) (mm)	0.90	0.64	2.13	0.00	0.85	(0, 2.15)
L(DL) (mm)	6.53	1.22	8.41	4.17	6.71	(4.14, 8.92)
WL(DL) (mm)	12.28	1.35	14.26	9.48	12.29	(9.63, 14.93)

*MD* crown mesiodistal diameter, *BL* crown buccolingual diameter, *H* crown height, *R* ratio of mesiodistal to buccolingual diameter, *h1* vertical distance from highest point of pulp chamber roof to occlusal surface, *h2* vertical distance from highest point of pulp chamber roof to midpoint of pulp chamber floor, *h3* vertical distance from midpoint of pulp chamber floor to midpoint of root bifurcation, *α* angle between root and z-axis, *l* observable canal length, *d* distance from canal terminus to apex, *L* predicted canal length (l + d); *WL* working length. Root data were distinguished by M (mesial root), D (distal root), P (lingual root). Canal data are distinguished by mb (mesiobuccal), ml (mesiolingual), d (distal), db (distobuccal), dl (distolingual), p (lingual). When the same letter was used for both root and root canal abbreviations, uppercase letters were used to denote roots, and lowercase letters were used to denote root canals in the manuscript

**Table 2:** Measurements for Type B PMFMs

Type B	Mean	Standard deviation	Max	Min	Median	95% CI
MD (mm)	8.71	0.24	9.02	8.29	8.72	(8.24, 9.18)
BL (mm)	7.73	0.22	8.11	7.43	7.66	(7.30, 8.16)
R	1.13	0.05	1.21	1.04	1.12	(1.03, 1.23)
H (mm)	6.89	0.34	7.50	6.28	6.84	(6.22, 7.56)
h1 (mm)	2.58	0.32	3.06	2.12	2.57	(1.95, 3.21)
h2 (mm)	3.63	0.72	4.88	2.75	3.78	(2.22, 5.04)
h3 (mm)	1.24	0.26	1.81	0.87	1.15	(0.73, 1.75)
α(M) (°)	16.26	1.37	17.93	14.01	15.58	(13.57, 18.95)
α(D) (°)	23.39	1.65	26.57	20.97	22.46	(20.16, 26.62)
l(mb) (mm)	8.06	1.07	8.89	5.77	8.53	(5.96, 10.16)
d(mb) (mm)	1.33	0.95	3.19	0.00	1.30	(0, 3.19)
L(mb) (mm)	9.38	0.53	10.21	8.81	9.25	(8.34, 10.42)
WL(mb) (mm)	15.67	0.48	16.22	14.61	15.75	(14.73, 16.61)
l(ml) (mm)	4.89	0.75	6.61	3.87	4.73	(3.42, 6.36)
d(ml) (mm)	1.30	1.03	3.05	0.00	1.35	(0, 3.32)
L(ml) (mm)	6.19	0.83	7.56	5.21	5.92	(4.56, 7.82)
WL(ml) (mm)	12.48	0.86	13.82	11.26	12.34	(10.79, 14.17)
l(d) (mm)	5.68	0.57	6.45	4.68	5.70	(4.56, 6.80)
d(d) (mm)	0.53	0.70	2.04	0.00	0.17	(0, 1.90)
L(d) (mm)	6.22	0.99	7.83	4.68	6.03	(4.28, 8.16)
WL(d) (mm)	12.50	0.98	14.07	10.77	12.68	(10.58, 14.42)

*MD* crown mesiodistal diameter, *BL* crown buccolingual diameter, *H* crown height, *R* ratio of mesiodistal to buccolingual diameter, *h1* vertical distance from highest point of pulp chamber roof to occlusal surface, *h2* vertical distance from highest point of pulp chamber roof to midpoint of pulp chamber floor, *h3* vertical distance from midpoint of pulp chamber floor to midpoint of root bifurcation, *α* angle between root and z-axis, *l* observable canal length, *d* distance from canal terminus to apex, *L* predicted canal length (l + d); *WL* working length. Root data were distinguished by M (mesial root), D (distal root), P (lingual root). Canal data are distinguished by mb (mesiobuccal), ml (mesiolingual), d (distal), db (distobuccal), dl (distolingual), p (lingual). When the same letter was used for both root and root canal abbreviations, uppercase letters were used to denote roots, and lowercase letters were used to denote root canals in the manuscript

**Table 3:** Measurements of Type D PMFMs

Type D	Mean	Standard deviation	Max	Min	Median	95% CI
MD (mm)	8.35	0.43	9.03	8.00	8.41	(7.51, 9.19)
BL (mm)	8.86	0.36	9.24	8.30	8.92	(8.15, 9.57)
R	0.94	0.03	0.98	0.89	0.94	(0.88, 1.00)
H (mm)	6.77	0.30	7.20	6.28	6.80	(6.18, 7.36)
h1 (mm)	2.80	0.33	3.25	2.36	2.66	(2.15, 3.45)
h2 (mm)	3.07	0.31	3.40	2.55	3.15	(2.46, 3.68)
h3 (mm)	1.06	0.08	1.20	0.97	1.04	(0.90, 1.22)
α(M) (°)	18.93	3.01	24.75	14.25	19.06	(13.03, 24.83)
α(D) (°)	27.00	2.23	29.66	22.27	27.62	(22.63, 31.37)
α(P) (°)	32.64	5.44	41.86	25.22	33.07	(21.98, 43.30)
l(mb) (mm)	9.32	0.30	9.74	8.91	9.39	(8.73, 9.91)
d(mb) (mm)	1.03	0.77	2.23	0.00	1.08	(-0.48, 2.54)
L(mb) (mm)	10.35	0.82	11.91	9.32	10.11	(8.74, 11.96)
WL(mb) (mm)	16.22	0.66	17.56	15.31	16.17	(14.93, 17.51)
l(ml) (mm)	5.50	0.80	7.03	4.79	5.32	(3.93, 7.07)
d(ml) (mm)	1.69	0.33	2.20	1.17	1.76	(1.04, 2.34)
L(ml) (mm)	7.19	0.66	8.20	6.34	7.07	(5.90, 8.48)
WL(ml) (mm)	13.15	0.90	14.48	11.79	12.97	(11.39, 14.91)
l(p) (mm)	7.31	0.92	8.75	5.81	7.13	(5.51, 9.11)
d(p) (mm)	1.31	0.37	1.88	0.77	1.33	(0.58, 2.04)
L(p) (mm)	8.61	0.63	9.53	7.69	8.66	(7.38, 9.84)
WL(p) (mm)	14.48	0.40	15.10	13.76	14.47	(13.70, 15.26)
l(d) (mm)	7.40	0.55	8.12	6.41	7.56	(6.32, 8.48)
d(d) (mm)	0.60	0.61	1.55	0.00	0.36	(-0.60, 1.80)
L(d) (mm)	8.00	0.84	9.45	6.41	8.07	(6.35, 9.65)
WL(d) (mm)	13.87	0.74	15.05	12.90	13.69	(12.42, 15.32)

*MD* crown mesiodistal diameter, *BL* crown buccolingual diameter, *H* crown height, *R* ratio of mesiodistal to buccolingual diameter, *h1* vertical distance from highest point of pulp chamber roof to occlusal surface, *h2* vertical distance from highest point of pulp chamber roof to midpoint of pulp chamber floor, *h3* vertical distance from midpoint of pulp chamber floor to midpoint of root bifurcation, *α* angle between root and z-axis, *l* observable canal length, *d* distance from canal terminus to apex, *L* predicted canal length (l + d); *WL* working length. Root data were distinguished by M (mesial root), D (distal root), P (lingual root). Canal data are distinguished by mb (mesiobuccal), ml (mesiolingual), d (distal), db (distobuccal), dl (distolingual), p (lingual). When the same letter was used for both root and root canal abbreviations, uppercase letters were used to denote roots, and lowercase letters were used to denote root canals in the manuscript

Regarding the total WL of PMFMs, Type A PMFMs showed mean values of 15.52 ± 1.05 mm for the mesiobuccal canal, 12.81 ± 1.06 mm for mesiolingual canal, 12.45 ± 1.05 mm for distobuccal canal, and 12.28 ± 1.35 mm for distolingual canal. In this type, the mesiobuccal canal had a significantly greater WL than the other canals (*p* < 0.05, Cohen’s d ranging from 2.57 to 2.92), while no significant differences were observed among the mesiolingual, distobuccal, and distolingual canals (*p* > 0.05) (Table 1). In Type B, the WLs of the mesiobuccal and mesiolingual canals did not differ significantly from those in Type A, and the distal canal showed no significant difference from either the distobuccal or distolingual canals of Type A (*p* > 0.05) (Tables 1 and 2). In Type D, the WLs of the mesiobuccal and mesiolingual canals were comparable to those in Type A (*p* > 0.05), whereas the distal canal exhibited a significantly greater WL than the distobuccal and distolingual canals of Type A (*p* < 0.05, Cohen’s d = 1.10, 0.98, respectively) (Tables 1 and 3). Within Type D, the WL of the lingual canal was significantly shorter than that of the mesiobuccal canal but greater than that of the mesiolingual canal (*p* < 0.05, Cohen’s d = 3.18,1.98, respectively), with no significant difference compared to the distal canal (*p* > 0.05) (Table 3).

Statistical analysis revealed no significant differences in the above measurements based on location (left or right) or sex (*p* > 0.05).

For all statistically significant comparisons, Cohen’s d values ranged from 0.98 to 3.18, indicating large effect sizes, which strongly supports our findings.

### Observation results of other anatomical details

**Fig. 8:**
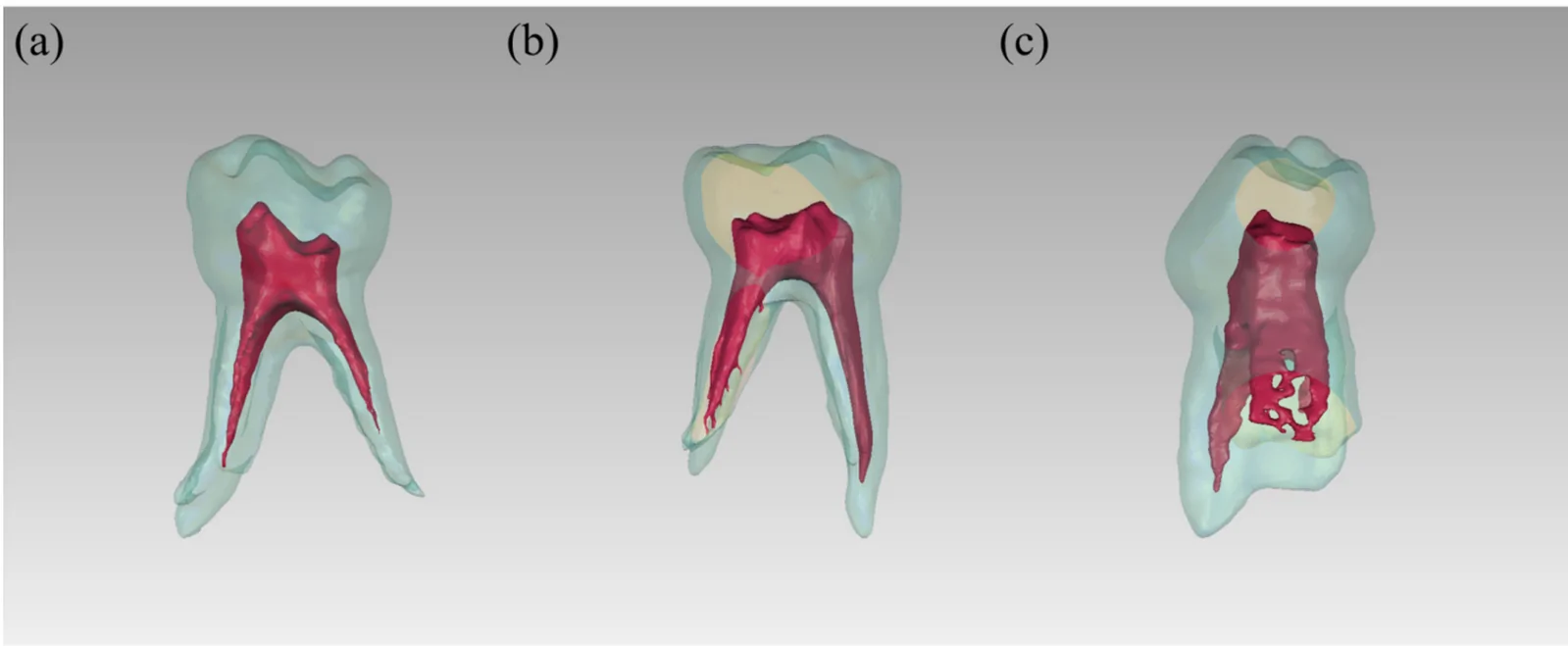
Other variations. **a** PMFM with outward curvature at the apex; **b** Lateral/accessory canal and apical delta; **c** Isthmus

In addition to the above classifications, PMFMs exhibited several other anatomical variations. In the apical third, some teeth showed root bifurcation, which generally corresponded to the canal trajectory; however, the apical foramen was not always located at the lowest point of the bifurcation. In some cases, the mesial and distal roots curved inward in the apical third, with corresponding curvature of the canals. In 4 Type A PMFMs (8.7%), either the mesial or distal root appeared straight in the middle and coronal thirds but curved outward in the apical third. In these cases, the canal did not follow the outward curvature but instead remained relatively straight, with the apical foramen located on the inner aspect of the root (Table 4; Fig. 8).

**Table 4:** Variation of roots in PMFMs

Root	Root variation		
	Bifurcation below apical third	Inward curvature	Outward curvature
Type A M	33 (71.7%)	16 (34.8%)	4 (8.7%)
Type A D	19 (41.3%)	5 (10.9%)	4 (8.7%)
Type B M	6 (50%)	5 (41.7%)	
Type D M	5 (71.4%)		
Type D D	3 (42.9%)		

Root data are distinguished by the annotations M (mesial root) and D (distal root)

In Types A, B, A1, and A2, variations such as lateral/accessory canals, isthmuses, and apical deltas were relatively common. In contrast, among three-rooted PMFMs, only one Type D1 tooth exhibited an isthmus in the distal root (Table 5; Fig. 8).

**Table 5:** Variation of root canals in PMFMs

Root	Root canal variation		
	Lateral/ accessory canal	Isthmus	Apical Delta
Type A M	9 (19.6%)	5 (10.9%)	2 (4.3%)
Type A D	12 (26.1%)	14 (30.4%)	10 (21.7%)
Type B M	2 (16.7%)	1 (8.3%)	
Type B D	2 (16.7%)	3 (25%)	2 (16.7%)
Type D1 D		1 (100%)	

Root data are distinguished by the annotations M (mesial root) and D (distal root)

## Discussion

A thorough understanding of the anatomy of primary teeth is essential for pediatric dentists, as it underpins the identification of physiological characteristics and pathological changes, as well as clinical management and education. However, previous studies have lacked detailed three-dimensional analyses of intact PMFMs [5–16]. In the present study, CBCT scans of 67 healthy, intact PMFMs were collected from 41 children in Beijing, China. These data were used to perform 3D reconstruction, detailed classification, and morphological measurements, revealing a potential association between crown morphology and root number.

This study has important implications for guiding the clinical management of PMFMs. First, it proposes a reproducible observational framework in which the cervical line and the line connecting the mesiobuccal and distobuccal cusps serve as references for model orientation, enabling consistent observation and measurements. This standardized approach helps reduce inconsistencies that may arise from variable landmark selection, thereby enhancing the reliability of morphological comparisons across different observers and studies.

Based on this framework, our findings demonstrate that the ratio of mesiodistal to buccolingual crown diameter (R) serves as a highly reliable predictor of root number. ROC curve analysis showed that *R* = 1.00 perfectly distinguished two‑rooted from three‑rooted teeth (AUC = 1.000, 100% sensitivity and specificity). Notably, this clear separation remained even when a single atypical three‑rooted variant (Type C, *R* > 1) was included in the analysis, indicating that the relationship between crown contour and root number is highly consistent across major morphological types. Specifically, when *R* > 1, the roots were predominantly mesiodistally aligned and two‑rooted; only one case (Type C) exhibited bifurcation of the mesial root into buccal and lingual branches. When *R* < 1, all samples showed three roots, including mesial, distal, and an additional lingual root, corresponding to the convexity on the lingual aspect of the crown. This consistent anatomical correspondence suggests that preoperative assessment of crown morphology may assist clinicians in predicting root configuration before endodontic or surgical procedures.

Notably, previous studies have rarely reported three-rooted PMFMs, and the reported incidence appears low. Ozcan et al. [6] reported a 5.56% (4/72) incidence of three-rooted PMFMs in Turkish children, whereas Moyaho-Bernal et al. [16] reported incidences of 0.44% in males and 0.22% in females in Mexican children. In the present study, the prevalence of three-rooted PMFMs was 11.9%. This discrepancy may be attributable to ethnic differences [6, 16]. Previous studies on permanent teeth have reported ethnic differences in the prevalence of three-rooted variants across different tooth types. In premolars—the successors of primary molars—three-rooted variants are more commonly observed in Caucasians, whereas three-rooted mandibular molars are more frequent in Mongoloid populations [24, 25]. Therefore, it is reasonable to infer that the observed differences in the prevalence of three-rooted PMFMs may also be related to ethnic background, and caution is warranted when generalizing these findings to other populations.

By integrating crown morphology, root number, and canal number, PMFMs were classified into seven types in this study. Teeth with *R* > 1 exhibited 3–5 canals, whereas those with *R* < 1 exhibited 4–5 canals, with four canals being the most common in both groups. In cases with *R* > 1, canal orifices were consistently located on the mesial and distal aspects of the pulp chamber floor along the buccolingual direction. In contrast, when *R* < 1, an additional canal orifice was typically located on the lingual aspect of the pulp chamber floor. It should be noted that, due to the limited sample sizes of certain rare subtypes (e.g., Types A1/A2, and C, D1), the related findings should be interpreted from a descriptive perspective and should not be generalized in terms of prevalence or morphological patterns.

Subsequently, detailed anatomical measurements were performed for tooth types with a sufficient sample size.

Among the two crown variants, the mesiodistal diameter did not differ significantly; however, the *R* < 1 group exhibited a significantly greater buccolingual diameter, suggesting that this variant may require preformed crowns with a different contour from those for the predominant type. In addition, crown measurements in this study were slightly larger than those reported in previous studies using 3D intraoral scans or two-dimensional measurements with vernier calipers [10–13]. This difference may be attributable to the difficulty of obtaining the maximum mesiodistal dimension near the contact area using those methods. These findings suggest that existing preformed crowns may require further optimization based on updated 3D data. In addition, anatomical variations across populations may necessitate the development of crown designs better adapted to population-specific morphological characteristics.

Previous studies have provided limited quantitative data on root canal anatomy in PMFMs, and most did not provide working length from the crown to the apex. For example, Fumes et al. [5] performed micro-CT analysis of 10 extracted PMFMs and reported root and canal length distributions. However, as the distance from the canal orifice to the apex is difficult to measure directly in clinical practice, the clinical applicability of these measurements is limited compared with WL. In that study, the mean mesial canal length was 6.1 ± 1.5 mm, and the distal canal length was 4.7 ± 2.5 mm, both of which are shorter than the corresponding canal lengths observed in the present study. This difference may be attributed to the use of healthy in vivo teeth in the present study, thereby avoiding the morphological alterations caused by caries or root resorption.

This study provides the average working length for each canal in Types A, B, and D PMFMs. Across these types, the mesiobuccal canal consistently exhibited the greatest WL, whereas no significant differences were observed among the remaining canals in Types A and B. In Type D, the WL of the mesiolingual canal did not differ significantly from those in Types A and B, while the distal and lingual canals were of similar lengths, slightly longer than the mesiolingual canal but substantially shorter than the mesiobuccal canal. These findings suggest that, in addition to comparing measured canal lengths with established averages, relative WL differences among canals within the same tooth may serve as useful internal references during clinical assessment. Additionally, this study identified several anatomical variations in PMFMs, including lateral and accessory canals, isthmuses, and apical deltas. These findings underscore the potential value of pulp-preserving approaches, such as pulpotomy, in cases with infected coronal pulp and healthy radicular pulp. They also highlight the importance of thorough chemical debridement and 3D obturation during root canal treatment.

This study has several limitations. First, the sample size was relatively limited, with an uneven distribution across morphological subtypes, particularly in the *R* < 1 group and less common canal configurations. This may have reduced statistical power and limits the robustness of subgroup analyses. Second, the sample was derived from a single institution in Beijing and represents a relatively homogeneous population, which may restrict the generalizability of the findings to other ethnic groups. Third, the retrospective use of CBCT data may introduce selection bias, as scans were obtained for specific clinical indications rather than from a population-based sample. Fourth, the inherent resolution limitations of CBCT may have reduced the detectability of fine anatomical structures such as accessory canals and apical deltas, potentially leading to underestimation of their prevalence. Finally, the classification system and the threshold value of the crown ratio (*R* = 1) were defined empirically based on this dataset and have not been externally validated. Given the exploratory nature of this study and the limited sample size for certain rare subtypes, our findings should be interpreted as preliminary and warrant further validation in larger, multicenter cohorts.

## Conclusion

This study presents a 3D anatomical characterization of PMFMs based on CBCT-derived models. The results demonstrate that crown morphology, particularly the mesiodistal-to-buccolingual ratio, may serve as a useful predictor of root number and distribution, with *R* < 1 strongly associated with the presence of an additional lingual root. PMFMs exhibited considerable variability in root canal morphology, with the four-canal configuration being the most common. The mesiobuccal canal consistently showed the greatest length across all variant types. These findings provide detailed anatomical data and may contribute to improving the precision and safety of clinical treatment.

## Data Availability

The data of this research are available upon reasonable request from the corresponding author.

## Abbreviations

PMFMs: Primary mandibular first molars
CBCT: Cone-beam computed tomography
3D: Three-dimensional
R: ratio of mesiodistal to buccolingual diameter
N: number of roots
n: number of Root canals
DICOM: Digital Imaging and Communications in Medicine
MD: crown mesiodistal diameter
BL: Crown buccolingual diameter
H: crown height
h1: vertical distance from highest point of pulp chamber roof to occlusal surface
h2: vertical distance from highest point of pulp chamber roof to midpoint of pulp chamber floor
h3: vertical distance from midpoint of pulp chamber floor to midpoint of root bifurcation
α: angle between root and z-axis
l: observable canal length
d: distance from canal terminus to apex
L: predicted canal length (l+d)
WL: working length
K-S: Kolmogorov-Smirnov
AUC: area under the curve
ROC: receiver operating characteristic
GLMM: generalized linear mixed model
